# Hepatocellular Carcinoma and Non-Alcoholic Fatty Liver Disease: A Step Forward for Better Evaluation Using Ultrasound Elastography

**DOI:** 10.3390/cancers12102778

**Published:** 2020-09-28

**Authors:** Monica Lupsor-Platon, Teodora Serban, Alexandra-Iulia Silion, Alexandru Tirpe, Mira Florea

**Affiliations:** 1 Medical Imaging Department, Iuliu Hatieganu University of Medicine and Pharmacy, Regional Institute of Gastroenterology and Hepatology, 400162 Cluj-Napoca, Romania; 2 Medical Imaging Department, Iuliu Hatieganu University of Medicine and Pharmacy, 400162 Cluj-Napoca, Romania; teodora.serban@elearn.umfcluj.ro (T.S.); alexandra.iuli.silion@elearn.umfcluj.ro (A.-I.S.); andrei.alex.tirpe@elearn.umfcluj.ro (A.T.); 3 Community Medicine Department, Iuliu Hatieganu University of Medicine and Pharmacy, 400001 Cluj-Napoca, Romania; miraflorea@umfcluj.ro

**Keywords:** hepatocellular carcinoma, non-alcoholic fatty liver disease, ultrasound elastography, fibrosis, steatosis, focal liver lesion

## Abstract

**Simple Summary:**

Non-alcoholic fatty liver disease (NAFLD) attracts a lot of attention, due to the increasing prevalence and progression to fibrosis, cirrhosis, and hepatocellular carcinoma (HCC). Consequently, new non-invasive, cost-effective diagnostic methods are needed. This review aims to explore the diagnostic performance of ultrasound (US) elastography in NAFLD and NAFLD-related HCC, adding a new dimension to the conventional US examination—the liver stiffness quantification. The vibration controlled transient elastography (VCTE), and 2D-Shear wave elastography (2D-SWE) are effective in staging liver fibrosis in NAFLD. VCTE presents the upside of assessing steatosis through the controlled attenuation parameter. Hereby, we critically reviewed the elastography techniques for the quantitative characterization of focal liver lesions (FLLs), focusing on HCC: Point shear wave elastography and 2D-SWE. 2D-SWE presents a great potential to differentiate malignant from benign FLLs, guiding the clinician towards the next diagnostic steps. As a disease-specific surveillance tool, US elastography presents prognostic capability, improving the NAFLD-related HCC monitoring.

**Abstract:**

The increasing prevalence of non-alcoholic fatty liver disease (NAFLD) in the general population prompts for a quick response from physicians. As NAFLD can progress to liver fibrosis, cirrhosis, and even hepatocellular carcinoma (HCC), new non-invasive, rapid, cost-effective diagnostic methods are needed. In this review, we explore the diagnostic performance of ultrasound elastography for non-invasive assessment of NAFLD and NAFLD-related HCC. Elastography provides a new dimension to the conventional ultrasound examination, by adding the liver stiffness quantification in the diagnostic algorithm. Whilst the most efficient elastographic techniques in staging liver fibrosis in NAFLD are vibration controlled transient elastography (VCTE) and 2D-Shear wave elastography (2D-SWE), VCTE presents the upside of assessing steatosis through the controlled attenuation parameter (CAP). Hereby, we have also critically reviewed the most important elastographic techniques for the quantitative characterization of focal liver lesions (FLLs), focusing on HCC: Point shear wave elastography (pSWE) and 2D-SWE. As our paper shows, elastography should not be considered as a substitute for FLL biopsy because of the stiffness values overlap. Furthermore, by using non-invasive, disease-specific surveillance tools, such as US elastography, a subset of the non-cirrhotic NAFLD patients at risk for developing HCC can be detected early, leading to a better outcome. A recent ultrasomics study exemplified the wide potential of 2D-SWE to differentiate benign FLLs from malignant ones, guiding the clinician towards the next steps of diagnosis and contributing to better long-term disease surveillance.

## 1. Introduction

Non-alcoholic fatty liver disease (NAFLD) has become a major public health issue, with a current global prevalence estimated at around 25%, and a tendency of rapidly growing [1]. The spectrum of NAFLD varies from simple steatosis to non-alcoholic steatohepatitis (NASH), but further progression can lead to fibrosis, cirrhosis, and hepatocellular carcinoma (HCC). A meta-analysis that included worldwide studies from 1985 to 2005 estimated the HCC incidence among NAFLD patients at 0.44 per 1000 person-years (range 0.29–0.66). Furthermore, the incidence of HCC in NASH was even higher, estimated at 5.29 per 1000 person-years (range: 0.75–37.56) [1]. At first sight, the NAFLD-related HCC incidence is low; however, the rise of concerns is given by the increasing prevalence of obesity worldwide, precisely a twofold growth in the last 40 years [2]. The prognosis of NAFLD-related HCC is poor and curative interventions are often excluded because of the late diagnosis. The etiologies for HCC in liver transplant candidates showed a shift during the last years, with a decrease of hepatitis C virus (HCV) and alcohol etiologies and a higher percentage of NAFLD-related HCC [3,4]. This trend highlights the increasing importance of detecting early developing HCC in NAFLD patients through rapid, non-invasive means.

Current guidelines lack recommendations for surveillance of non-cirrhotic NAFLD/NASH patients who are at risk for developing HCC. In a United States population-based study, 54% of patients were found to have NAFLD-related HCC without underlying cirrhosis [5]. A study by Mittal et al. [6] found that the NAFLD patients are five times more likely to develop HCC in the absence of cirrhosis than HCV patients. Notably, most NAFLD patients included in this study suffered from obesity and diabetes, supporting the pathogenetic hypothesis. Despite the poor prognosis of NAFLD-related HCC, due to late diagnosis and curative interventions often excluded, the recommendation on HCC surveillance in patients without significant fibrosis is controversial because of its low cost-effectiveness [7].

At the moment, ultrasound (US) is the first-line imaging method used for the screening of liver cancer, but the technique encounters several limitations in NAFLD patients. A study that aimed to investigate the drawbacks of US when detecting HCC estimated a US sensitivity in obese patients of 76% versus 87% in non-obese patients (*p* = 0.01). The same study found a US sensitivity of 59% for NASH detection versus 84% in the case of other etiologies of liver disease (*p* = 0.003). These results illustrate that the body mass index (BMI) and steatosis itself represent two independent factors leading to an inadequate ultrasound image [8]. Moreover, computed tomography (CT) and magnetic resonance imaging (MRI) scans are not affected by these US limitations; however, because of the radiation exposure and the high cost, respectively, these techniques may not be an appropriate choice for initial surveillance. Furthermore, the imaging diagnostic criteria for HCC detection on CT and MRI should be used with great carefulness in patients with underlying NASH, since 40% of HCC nodules do not display wash-out on the portal or delayed phase images on MRI; encapsulation was identified only in 60% of HCC nodules, leading to difficult interpretation [9,10]. Therefore, these patients are more likely to require a liver biopsy, which is able to confirm de HCC and characterize the status of the liver parenchyma affected by NAFLD [11]. The procedure is indicated with caution, as it holds the potential for severe complications and sampling errors [12]. In this regard, every novel information provided by noninvasive methods of evaluation can help the clinician run an early and accurate diagnosis and reduce the number of liver biopsies.

Recently, the ultrasound elastography has received widespread attention by adding a new dimension to noninvasively, easily accessible methods of assessing liver diseases. All liver diseases, focal and diffuse, are associated with changes in the structure of the tissue, with altered liver stiffness (LS), precisely the changes that elastographic techniques can detect and quantify. As such, this review aims to highlight the role of ultrasound elastographic techniques to assess both the focal liver lesions (FLLs) and the liver parenchyma status on which the FLL has developed.

## 2. Clinical Considerations: The HCC-NAFLD-NASH Trio

**Figure 1:**
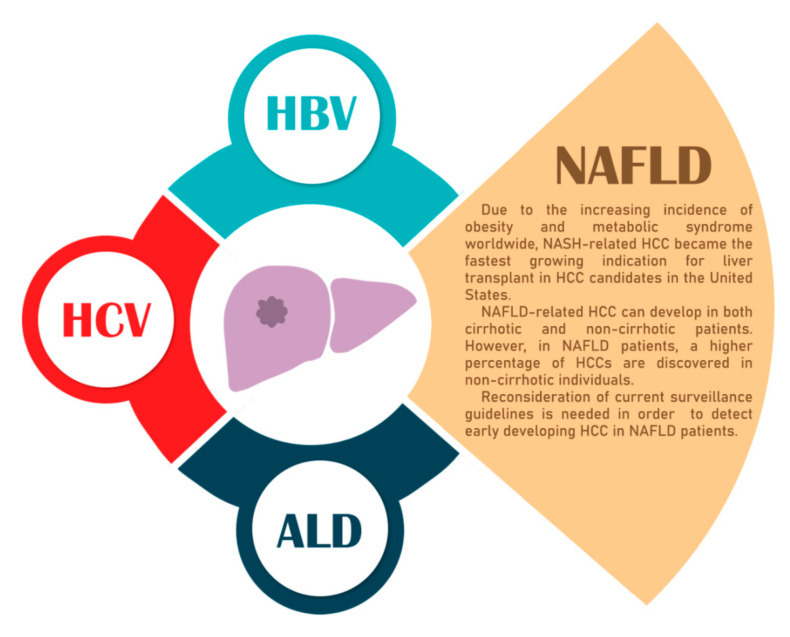
A graphical representation of the main hepatocellular carcinoma (HCC) etiologies. The main risk factors in HCC development are HBV, hepatitis C virus (HCV), alcoholic liver disease (ALD), and non-alcoholic fatty liver disease (NAFLD). The increasing prevalence of NAFLD and its silent progression towards fibrosis, cirrhosis, and HCC requires new non-invasive diagnostic methods. NASH, non-alcoholic steatohepatitis.

The spectrum of NAFLD varies from simple fatty liver, defined as triglyceride accumulation in more than 5% of the hepatocytes, to NASH, with the latter also including ballooning and lobular inflammation at the histological level. The NASH inflammatory state usually progresses with severe liver cell damage and subsequent fibrosis [13]. Concomitantly, NAFLD can progress with fibrosis as well. The further advancement of the disease can lead to serious consequences, such as compensated and decompensated cirrhosis and a higher risk of HCC [14]. We note that the NAFLD-related HCC can arise on both cirrhotic and non-cirrhotic livers, but a higher percentage has been reported in non-cirrhotic patients [5,15]. Figure 1 presents the main HCC etiologies, with a focus on NAFLD.

The increasing incidence of obesity and metabolic syndrome (MetS), along with their hepatic involvement–NAFLD–led to a change in the etiology of HCC. Furthermore, the improvements in the prevention and treatment of hepatitis B virus (HBV) and the current effective curative treatment of HCV indicate this transition as well.

The liver oncogenesis in NAFLD/NASH is complex and multifactorial, involving lipotoxicity, intestinal microflora dysregulation with elevated levels of lipopolysaccharide (LPS), hyperinsulinemia with insulin resistance, increased IGF levels, and a low grade chronic systemic inflammatory response [16]. Genetic polymorphism and increased iron absorption may be contributing factors for the NASH-related HCC development [17]; oncogenic mechanisms, such as telomere erosion, chromosome segregation defects, and alterations in the DNA-damage-response pathways, leading to genomic instability have been incriminated as well [18,19]. Kanwal et al. [20] reported that male sex, diabetes, and senior age are all independent risk factors for developing cancer. Additionally, Yang’s novel study [21] found that low albumin significantly predicted the development of HCC; whereas, body mass index (BMI), hypertension, and hyperlipidemia did not. In comparison, the hepatocarcinogenesis in HBV and HCV infections are associated with the induction of chronic inflammation, along with molecular alterations that may induce mutagenesis in the case of HBV. The expression of viral proteins and the viral life cycle are other factors that drive the carcinogenesis in these patients [22].

Moreover, several studies illustrated the high risk of HCC in cirrhotic NAFLD patients; in this particular case, bi-annual ultrasound (US) surveillance is universally recommended [14,23]. However, there are no clear guidelines for cost-effective surveillance of non-cirrhotic NAFLD patients [24]. Furthermore, there is compelling evidence that NASH-related HCC patients receive less surveillance and treatment compared to patients with other HCC etiologies [25]. Although patients with NAFLD/NASH usually present less aggressive HCCs, the likelihood that they may be diagnosed by current monitoring is low, leading to late diagnosis and a poor prognosis [26,27,28].

Reconsideration of the current surveillance guidelines is needed [29], to increase the detectability rate of HCC in NAFLD patients at a screening level. Using new imaging techniques, such as US liver elastography, combined with non-invasive biomarkers, a subpopulation of the non-cirrhotic NAFLD patients carrying a clinical risk of HCC development could be identified, leading to increased and early HCC detectability [30].

## 3. The Main Elastographic Techniques

Historically, elastography drew inspiration from the diagnostic palpation, a clinical method used to determine the consistency of an organ or a lesion. This technique is based on the elastic properties of the material, the ability to regain its shape and dimensions after being the subject of a deforming force [31]. Therefore, elastographic methods involve applying mechanical stress on a tissue and evaluating its behavior.

The most notable elastographic techniques use ultrasound or magnetic resonance imaging (MRI). Magnetic resonance elastography (MRE) has several strengths compared to the ultrasound techniques. MRE generates a quantitive 3D elasticity map that covers an entire organ, it is less operator-dependent, and it is not limited by air or bone. However, when considering liver assessment, MRE has a rather limited utility compared to the ultrasound methods because of the high costs and the limited availability [32]. This review will focus on the role of ultrasound elastographic techniques, due to their larger accessibility and potential to detect focal liver lesions.

According to several elastography guidelines [33,34,35], the ultrasound elastographic techniques can be classified as either quantitative (“Shear Wave Elastography”, SWE) or qualitative (“Strain Elastography”). The “strain” techniques are less used in the assessment of diffuse liver diseases. Currently, there are three main quantitative techniques used in clinical practice [35]:

- Vibration controlled transient elastography—VCTE (FibroScan^®^, Echosens, Paris, France)
- Point Shear wave elastography (ARFI-based technique): VTQ (Siemens Healthcare, Berlin, Germany), ElastPQ (Philips Healthcare, Amsterdam, The Netherlands), SWM (Hitachi Aloka Medical, Tokyo, Japan), QelaXto^®^ (Esaote, Genoa, Italy), S-shearwave^®^ (Samsung, Seoul, South Korea), STQ^®^ (Mindray, Shenzhen, China)
- 2D-Shear wave elastography (ARFI-based technique): SSI (SuperSonic Imagine, Aixplorer^®^, Aix-en-Provence, France), ElastQ^®^ (Philips, Healthcare, Amsterdam, The Netherlands), 2D-SWE.GE (GE Healthcare, Chicago, IL, USA), ToSWE (Toshiba Medical Systems, Tokyo, Japan), STE^®^ (Mindray, Shenzhen, China)

### 3.1. Vibration Controlled Transient Elastography (VCTE)

VCTE is an elastographic technique that displays the shear wave velocity through the liver; the FibroScan device [34] consists of an ultrasonic transducer mounted at the end of an electrodynamic vibrator [36,37]. A single cycle of low-frequency vibrations (50 Hz) is applied at the surface of the body, producing a transient shear wave deformation, which propagates deeper in the liver parenchyma. The shear wave is tracked through multiple ultrasounds acquisitions, whilst the speed of the wave is calculated and used to deduct the Young modulus, according to the equation E = 3ρVs2, where E is the Young or the elasticity modulus, ρ is the density of the material (constant), and Vs2 is the velocity of the shear wave previously determined [32,38,39]. Young modulus, E, is measured in kilopascals (kPa), and it corresponds to the liver stiffness, such that a higher value indicates a stiffer tissue. Liver stiffness range between 1.5 to 75 kPa [40,41] with normal values at around 4.5 to 5.5 kPa in the healthy population [35]. The equipment displays the median of the measured Young’s modulus, the interquartile range (IQR), the interquartile range/median ratio (IQR/M) after 10 conclusive measurements, and also the success rate of the measurements (SR) [31]. According to the European Federation of Societies for Ultrasound in Medicine and Biology (EFSUMB) guideline, it is necessary to obtain 10 valid measurements with an IQR < 30% of the median value of the liver stiffness for a good test reliability [35].

One of the major drawbacks of VCTE is the low success rate among obese subjects [42]. Because of that, the manufacturer created a new XL probe destined to high BMI subjects that is able to increase the VCTE success rate. As such, the M probe is used for standard examinations, and the XL probe is designed to evaluate overweight patients. Using a lower frequency of 2.5 MHz, the XL probe allows liver stiffness measurement at a greater depth, being more reliable than the regular (M) probe when used in patients with BMI > 30 kg/m^2^ [43,44,45].

Furthermore, VCTE is able to evaluate a predefined volume of approximately 1 cm wide per 4 cm long cylinder—at least 100 times the size of a biopsy sample. Consequently, the method not only makes a non-invasive assumption of the fibrosis and steatosis status, but the sample volume is considerably larger when compared to the standard biopsy [31].

#### Controlled Attenuation Parameter—A Novel Tool for Steatosis Assessment Using VCTE

The Controlled Attenuation Parameter (CAP) estimates the total ultrasonic attenuation and has been developed as a feature of the FibroScan^®^ device for assessing liver steatosis [46,47]. CAP is evaluated using the same radiofrequency data, and the same region of interest as the region used to assess the LS. Therefore, the equipment can measure the liver stiffness (for the estimation of fibrosis) at the same time with CAP (for the estimation of steatosis) [39,43,46,48]. CAP is expressed in dB/m, ranging between 100 and 400 dB/m, with normal values under 247 dB/m [49]. As CAP was first implemented on the M probe, the add-on of CAP on the XL probe overcame the measurement failure acquired in 7.7% of cases when using the standard M probe in patients with increased BMI (>30 kg/m^2^) [50]. Both probes have similar diagnostic performance, and recent studies have shown similar cut-offs when used accordingly to each patient’s morphology [51,52,53].

### 3.2. Point Shear Wave Elastography (pSWE)

“Point SWE” is another category of elastographic techniques; our review will focus on the acoustic radiation force impulse (ARFI) technique (Siemens), since it is, to date, the only one that has been analyzed in the context of NAFLD patients. This quantitative technique provides a single uni-dimensional measurement of tissue elasticity, similar to FibroScan^®^. Furthermore, the 1 × 0.5 cm measurement area can be positioned by the evaluating physician on a two-dimensional bright-mode (B-mode) US image in any region of the hepatic parenchyma with no vasculature and to a maximum depth of 8 cm from the skin plane. Point shear wave elastography (pSWE) measures the shear wave velocity (SWV), in m/s, that was induced by the acoustic radiation propagating in the tissue [39,54,55,56,57]. The normal values range between 1.01 m/s and 1.59 m/s in healthy individuals [35].

### 3.3. Two-Dimensional SWE (2D-SWE)

“2D-SWE” is another category of US quantitative elastographic technique. Compared to the pSWE technology, which produces displacement in a single focal location, the 2D-SWE produces dynamic stress in multiple focal zones, using the same ARFI technique. The shear waves propagate laterally in the shape of a cone, and the ultrasound detection pulses provide acquisitions at a very high rate to detect the movement in real-time and to evaluate the SWV [31,35]. The Young’s modulus (E) is determined by the equation E = 3ρc^2^, where ρ is the tissue density (constant), and c is the shear wave speed [35]. A colored map of the stiffness is created and is superimposed on the B-mode image of the ultrasound equipment, providing both anatomical (ultrasonic) and stiffness information. The quantitative assessment of the stiffness is also available, and the results are provided in kPa or m/s [58,59]. In healthy people, the Young’s modulus varies between 4.5 to 5.5 kPa using the SuperSonic Imagine (SSI) equipment [35], which is the most validated system in liver pathology among those that have 2D-SWE. Several advantages can be highlighted: The ROI has the adjustable size, and is larger than the ROI provided by pSWE; the method is ultrasound-guided and has real-time visualization. It is worth mentioning that this technique is feasible in patients with ascites or obesity.

### 3.4. Strain Elastography (SE)

SE has the lowest applicability for liver evaluation. It involves mechanical stress produced by either palpation with the ultrasound transducer or by physiological movements (heart beats, respiratory movements). The axial displacement is relative to the surrounding tissue and is compared before and after the pressure is applied. With SE, there is substantial variability of the results, due to the inability to quantify the stress and the consequent relative deformability [31,35,60]. This feature is considered a major drawback that limits the use of SE in accurately evaluating diffuse liver diseases. However, SE can be used in the qualitative evaluation of FLL by characterizing the lesion as either soft or hard.

## 4. Confounders: Factors Influencing Liver Stiffness Independent of Liver Fibrosis

In general terms, the main confounders of elevated liver stiffness are the same for all techniques. They include necroinflammation, congestion, and mechanic cholestasis. Food intake and alcohol consumption can also influence the results. Other diseases that can independently increase the liver stiffness are amyloidosis, lymphomas, and extramedullary hematopoiesis [35,61].

In addition, the software may represent a source of measurement bias. Measured cut-off values are specific for each manufacturer and for each product of the same brand. For this reason, the data provided by different manufacturers should not be pooled together. Therefore, it is recommended to consider the data from a single product to have interobserver comparability [35].

Another disputed error point is whether severe steatosis influences liver stiffness. Petta et al. [62] found that severe steatosis (≥66% at liver biopsy and severe bright liver ultrasound pattern) significantly increases LS values by using the M probe in NAFLD patients. They assumed that the fat droplets in the hepatocytes alter the wave transmission through the liver, increasing the rates of false-positive diagnoses of both significant and severe fibrosis. More recently, similar results for high CAP values by the regular M probe were reported [63]. However, it is debatable whether steatosis directly affects fibrosis measurement. Several studies showed that high BMI and central obesity were independent risk factors for liver stiffness measurement (LSM) unreliability and for a high rate of failure [42,64]. A novel prospective study by Wong et al. [65], found that BMI rather than steatosis was a more important confounder of fibrosis assessment in NAFLD patients. Nevertheless, further studies are required to elucidate this aspect.

## 5. Indications, Advantages, and Limitations of the Quantitative Ultrasonic Elastography Technology

The main clinical indications for ultrasound elastography in patients with chronic liver diseases are detection, staging, and monitoring liver fibrosis [38]. As exemplified in Table 1, all elastographic techniques showed promising results in patients with HCV. With a range of 56–100% sensitivity and 32–98% specificity pooled in the European Association for Study of Liver-Asociacion Latinoamericana para el Estudio del Higado (EASL-ALEH) Clinical Practice Guidelines [35], these techniques are being considered as a first-line assessment for liver fibrosis by current protocols [35]. Similarly, in patients with HBV, elastographic methods proved their usefulness in identifying those with cirrhosis with a sensitivity range of 50–100% and a specificity of 38–98%. On the other hand, studies concerning NAFLD subjects are rather scarce, with a large amount of the literature focusing on VCTE, since pSWE and 2D-SWE are newer technologies. Of note is that the control patients in NAFLD studies are individuals with no underlying liver disease. A more comprehensive approach to NAFLD assessment will be presented in the sections below.

## 6. Liver Parenchyma Characterization in NAFLD Patients with Superimposed HCC

As NAFLD statistics increase worldwide, it is imperative to identify those with unfavorable prognosis and implement repeatable, non-invasive methods for proper assessment and screening. Elastographic techniques, such as VCTE, pSWE, and 2D-SWE, are recent developments that can accurately evaluate liver stiffness. In general terms, the stiffer the tissue—the greater the amount of liver fibrosis. Concomitantly, liver steatosis can be easily evaluated through VCTE by measuring the aforementioned CAP.

### 6.1. Performance of VCTE for Liver Fibrosis Assessment in NAFLD

VCTE is a noninvasive, easy-to-perform method that can reliably determine the stage of liver fibrosis in patients with NAFLD by measuring liver stiffness [75,76,77]. As VCTE presents a high negative predictive value (around 90%), it can be used with great confidence to exclude severe fibrosis and especially cirrhosis, rather than diagnosing these pathological entities [35,78,79].

For several years, numerous studies reviewed the performance of liver stiffness measurement assessed by VCTE, compared to the histological evaluation through liver biopsy, which is considered to be the “gold standard” for fibrosis assessment [35]. As summarized in Table 2, the diagnostic cut-off values for minimal fibrosis (≥F1) using the M probe range from 4.9 to 7 kPa, with 61.7–90% sensitivity and 31–100% specificity. The proposed cut-offs for diagnosing advanced significant fibrosis (≥F2) with the same probe vary between 5.8 to 12.1 kPa, with 40–91.7% sensitivity and 38–94.4% specificity. Moreover, the vast majority of studies that analyzed the cut-off values for detecting severe fibrosis (≥F3) found suggestive LS values of 6.2 to greater than 15 kPa with 28.6–100% sensitivity and 47–98.7% specificity. As expected, the cut-off values for liver cirrhosis are high, varying from 7.9 to 22.3 kPa with 46.9–100% sensitivity and 62–98% specificity, using the M probe. Furthermore, the meta-analysis performed by Xiao et al. [80] recommends 4.8 to 8.2 kPa as the threshold for ruling in stage 2 fibrosis with the new XL probe at an associated 75.8% sensitivity and 64.8% specificity; the same meta-analysis suggests a range between 5.7 to 9.3 kPa for stage 3 fibrosis with 75.3% sensitivity and 74% specificity [80]. The Xiao meta-analysis propounds cut-off values varying between 7.2 to 16 kPa for cirrhosis with 87.8% sensitivity and 82% specificity, when the XL probe is used. Overall, in our reviewed studies, the AUROC ranged from 0.74 to 0.93 for stage 1 fibrosis, 0.757 to 0.987 for stage 2 fibrosis, 0.76 to 0.98 for stage 3 fibrosis, and 0.836 to 0.99 for stage 4 fibrosis. 

#### VCTE—Impediments and Resolutions

The main challenge with the VCTE technique is to obtain valid acquisitions in high BMI patients, as abdominal obesity hampers the transmission of the shear wave [35,93]. Different studies have reported unreliable results (11.6–15.8%) and a high rate of failure (2.7–23%), mostly because of increased BMI (≥28 kg/m^2^) along with elevated waist circumference [42,43,76,79,91,92,94,95]. Other features of the metabolic syndrome, together with limited operator experience, correlate with measurement failure [94]. These findings strengthen the need to validate the new XL probe, designed for obese patients, that should be used when the skin-to-liver capsule distance (SCD) is greater than 25 mm [34]. Multiple studies reported that when used in the same patient, the XL probe generates lower measurement than the M probe. Therefore, it has been thought that the cut-off values for the XL probe should be lower, around 1.5–2 kPa, than the ones used for the standard M probe [35,40,68,96,97]. However, in a novel prospective study [65], Wong et al. found that the same LS cut-off values can be used for both M and XL probe in clinical practice, when used in patients with BMI <30 kg/m^2^ and ≥30 kg/m^2^, respectively, as the high BMI independently increases liver stiffness values [98].

### 6.2. pSWE Performance in Assessing Fibrosis in NAFLD

We identified several studies that assessed point shear wave elastography-ARFI in NAFLD patients [87,99,100,101,102,103]. A systematic review and meta-analysis by Liu et al. [104] found that ARFI elastography has modest accuracy (about 90%) in detecting significant fibrosis in NAFLD patients, with 80.2% sensitivity and 85.2% specificity. These values are considered an inappropriate endpoint by the ESFUMB guidelines [35]. However, Friederich-Rust et al. [68] found that ARFI has similar diagnostic accuracy to VCTE in detecting significant and severe fibrosis, in line with the results of the meta-analysis conducted by Jiang et al. [84]. In a novel 2020 systematic review and meta-analysis by Lin et al. [105], which included 1147 NAFLD patients, the AUROC was 0.89, 0.94, and 0.94 for the diagnosis of stages 2, 3, and 4 of fibrosis, respectively. Considering these contradictory results, further longitudinal studies should clarify its performance for monitoring patients with NAFLD. Overall, the AUROC systematized in Table 3 ranges from 0.657 to 0.944 for advanced fibrosis, 0.71 to 0.982 for severe fibrosis, and 0.74 to 0.984 for cirrhosis prediction.

Compared to other elastographic techniques, several studies investigated the ability of ARFI to distinguish between patients with NASH from those with simple steatosis, concluding that pSWE is a promising tool with AUROC varying from 0.867 to 0.899 [101,102].

### 6.3. Performance of 2D-SWE in Evaluating Fibrosis in NAFLD Patients

2D-Shear Wave Elastography is a relatively new FDA-approved technique that measures liver stiffness using acoustic radiation force and ultrafast ultrasound imaging [87], with limited research on the diagnostic accuracy in NAFLD. Two meta-analyses [106,107] that included 2303 and 934 patients with chronic liver diseases, respectively, evaluated the performance of 2D-SWE in assessing liver fibrosis. The pooled sensitivity and specificity of SWE were 76% and 92% for ≥ F1, with an AUROC of 0.85. The summary AUROC was 0.87–0.88 for ≥ F2 with a sensitivity of 84–85% and a specificity of 81–83%. For ≥ F3 the pooled sensitivity and specificity were 89–90% and 81–86%, respectively, corresponding to an AUROC of 0.93–0.94. The pooled sensitivity and specificity for ≥ F4 were 87–88% and 88–89%, with AUROC 0.92–0.94.

Furthermore, recent studies on NAFLD patients suggest that this elastographic method achieved good diagnostic performance, with AUROC values ranging from 0.75 to 0.89 for ≥ F2, 0.8 to 0.95 ≥ F3, and 0.88 to 0.97 for F4, being particularly useful in detecting lower stages of fibrosis with AUROC values of 0.82 for ≥ F1 [108,109], as exemplified in Table 4. Regarding the cut-off values for different fibrosis stages, Cassinotto et al. [87] showed that most of them are very close to the corresponding VCTE values for ruling out the pathologies

### 6.4. Steatosis Evaluation in NAFLD Patients Using the Controlled Attenuation Parameter (CAP)

VCTE is able to measure the LS and CAP simultaneously [40]. The latter evaluates the amount of liver steatosis, defined as fat accumulation in the hepatocytes, the only histopathological factor that influences this parameter [115,116,117]. In comparison, the conventional B-mode US provides a subjective estimation of fatty infiltration and is mostly unreliable in detecting mild steatosis [118].

A 2016 meta-analysis involving 2735 patients (with a 20% intra-study prevalence of NAFLD) provided the optimal CAP cut-off values of 248 dB/m, 268 dB/m, and 280 dB/m for the prediction of mild, moderate, and severe steatosis, respectively. According to this meta-analysis, covariates, such as etiology, BMI, and diabetes, should be taken into consideration when interpreting CAP, although sex, age, and fibrosis have been shown to play a rather minor role. The authors recommend using the aforementioned cut-off values, but deducting 10 dB/m from the CAP value for NAFLD/NASH patients, 10 dB/m for diabetes patients, and deducting/adding 4.4 dB/m for each unit of BMI above or below 25 kg/m^2^ and over the range of 20–30 kg/m^2^ [49].

Furthermore, in a recent meta-analysis by Pu et al. involving 1297 biopsy-proven NAFLD patients, the mean AUROC value of CAP was 0.96, 0.82, and 0.70 for diagnosing mild, moderate, and severe steatosis, respectively [119]; the Pu study did not provide any cut-off values for NAFLD patients.

### 6.5. Prognosis Value of LS and CAP Measurement in NAFLD

It is imperative to assess the fibrosis stage in NAFLD patients, as it represents the key prognostic factor for liver-related events [120,121,122]. Mortality rises by a factor of 50–80 for NAFLD patients with severe fibrosis (F3) or cirrhosis (F4) compared to those with mild or no fibrosis [13]. In a retrospective cohort study on 646 biopsy-proven NAFLD patients, Hagström et al. [123] found that NASH did not affect the outcomes of patients in a significant manner, whereas, higher stages of fibrosis did.

In a recent prospective study on 2551 NAFLD patients, Shili-Masmoudi et al. [124] demonstrated that LS is an independent predicting factor for overall survival, liver-related and cardiovascular events, supporting the meta-analysis findings of Singh et al. [125]. Shili-Masmoudi also showed that the HCC incidence rises with baseline LS from 0.32% (if LS < 12 kPa) to 0.58% (if LS ranges between 12–18 kPa), 9.26% (if LS ranges between 18–38 kPa) and 13.3% (if LS >38 kPa) [124]. Moreover, several studies established the association between LS and the risk of HCC development in patients with chronic hepatitis C [126,127,128] and chronic hepatitis B [129,130], providing effective risk prediction models [131,132,133]. However, existing literature does not offer any model for NAFLD-related HCC risk.

Boursier et al. [86] evaluated the prognostic significance of LS in NAFLD, recommending a new clinically relevant fibrosis classification using seven classes of fibrosis: LSM1 (between 2.0 and 4.6 kPa), LSM2 (4.6 to 6.1 kPa), LSM3 (6.1 to 8.8 kPa), LSM4 (8.8 to 12.0 kPa), LSM5 (12.0 to 18.0 kPa), LSM6 (with a large interval between 18.0 to 38.6 kPa) and LSM7 (when liver stiffness is greater than 75.0 kPa). In the Boursier study, overall survival progressively decreased with increasing LS. For instance, overall survival for LSM1 in ten years was close to 1.0, indicating almost perfect concordance; whereas, for LSM7, the Harrel-C index was near 0.3 [86].

Regarding the prognostic value of CAP, studies are rather scarce and have conflicting results. Margini et al. reported that a CAP > 220 dB/m was independently associated with a higher risk of relevant clinical events [134]. On the other hand, Liu et al. reported that neither the presence nor the severity of liver steatosis as measured by CAP forecasted cancer, liver-related or cardiovascular events [135]. These results are in line with the latest results of Scheiner and colleagues [136]. Therefore, further research is necessary to elucidate the prognostic role of CAP among NAFLD patients.

## 7. Ultrasound Elastography: A New Tool in the Characterization of Hepatocellular Carcinoma in Non-Alcoholic Fatty Liver Disease

As exemplified so far, elastography is a powerful non-invasive diagnostic tool used in a number of diffuse liver diseases, including NAFLD. In addition, ultrasound elastography is able to characterize focal liver lesions (FLLs), providing supplementary information to the diagnostician.

In the context of NAFLD, elastography may play an important role in differentiating HCC, a known complication of this disease, from other focal liver lesions. Of note is the high incidence of HCC that arises from a NAFLD-affected liver in the absence of fibrosis or cirrhosis [9]. Indeed, it is abundantly clear that an in-depth stiffness measurement of the FLL should invariably be associated with the elastographic evaluation of the background liver. It is worth mentioning that there is a large FLL stiffness value overlap between benign and malignant FLL, which limits the accurate use of elastography for the diagnosis of a specific FLL in this type of patient.

From a technical perspective, VCTE is not able to characterize the stiffness of a single FLL. Of note are several studies that investigated the role of VCTE for HCC prediction in cirrhotic patients of specific viral etiology, or to correlate liver stiffness measurements with survival and prognosis; these studies identified a statistically significant correlation between a higher liver stiffness baseline value and the risk of developing HCC in patients with B and C chronic viral hepatitis [125,129,137,138]. We found no studies that focused on the diagnostic capability of VCTE in NAFLD-related HCC.

### 7.1. The Evaluation of FLLs Using pSWE Methods

Considering that pSWE is a noninvasive and reproducible method that can be used in liver fibrosis assessment, several recent studies sought to investigate pSWE performance for FLL evaluation, with a target to differentiate the large number of FLLs and to characterize their cancerous/benign state [139,140,141,142,143,144,145,146,147,148,149,150,151,152,153,154].

ARFI measures of FLLs are best interpreted in the context of the liver background, as it may suggest an FLL on diffuse liver disease. We reiterate the idea that HCC can arise on several altered liver backgrounds, such as cirrhotic livers of different etiologies and even NAFLD-affected livers. Table 5 presents a collection of shear wave velocity values (mean in m/s, range) measured by pSWE in different types of FLLs: HCC, metastases, hemangiomas, focal nodular hyperplasia (FNH), and adenomas, as well as the corresponding SWV cut-off values (m/s) for discriminating between the malignant versus benign FLL status. Literature data suggest that malignant FLLs are generally stiffer than their benign counterparts [152,155]; HCCs are overall softer than other malignant tumors [140,141,143], with SWS values varying from 2.17 ± 0.85 m/s in the Gallotti study [143] to 3.07 ± 0.89 m/s in the Guo study [146]. Several elastographic FLL studies report the following descending order of stiffness, based on the ARFI method: Metastases > HCC > FNH > hemangiomas [140,147,148]. Of note is the SWV value similarity between different pathological processes, such as between HCC and FNH [147,148] and even between the malignant category and the benign category in the Dong study [145]. A plausible explanation for these overlaps include the level of fibrous tissue in the focal lesion, as well as the vascularization; whilst fibrous tissue tends to increase stiffness, highly vascularized lesions tend to be softer [152]. These factors may limit the diagnostic capability of ARFI for the precise diagnostic of the FLL. Nevertheless, several studies concluded that pSWE presents promising utility in discriminating between HCC versus other FLLs [156].

pSWE evaluation of FLLs has several limitations and error points that are worth discussing. First and foremost, the maximum depth of pSWE examination is limited to 8 cm from the skin, due to safety concerns [157,158]; therefore, lesions situated below 8 cm cannot be examined. Another pSWE limitation relates to the susceptibility of motion-related factors that can lead to an inaccurate reading of the SWV; the inaccuracy increased when the focal lesion was located close to the heart or large blood vessels, as well as in patients unable to keep the breath-hold [159]. Furthermore, the wide range of stiffness values/SWVs of FLLs leads to value overlapping between malignant and benign lesions, leading to a diagnostic confusion [144]. Sampling bias is another error point that is worth mentioning [152]; Frulio et al. suggested that different measurement findings in studies that compare benign versus malignant lesions can be explained by different proportions of these FLLs in the study samples. For example, a significantly larger number of patients with FNH could increase the mean SWV value of the benign FLLs group, as was the case in the same study by Frulio et al. [144]. Last, but not least, we mention the limitations that may arise regarding the study design (inclusion/exclusion criteria) and the operator’s experience. Nevertheless, ARFI still remains a powerful and essential diagnostic tool in the differential evaluation of FLLs.

### 7.2. 2D-SWE Evaluation of FLLs

2D-SWE has been used in multiple clinical instances, such as discriminating with high specificity between malignant and benign lesions in the prostate [160], thyroid [161], breast [162], and more recently, for the non-invasive characterization of focal liver lesions [163,164]. Compared to pSWE, 2D-SWE supersonic shear imaging allows the display of color maps with quantitative data [165], further enlarging the information palette that elastography could potentially bring in the diagnosis of HCC in NAFLD. There are studies that evaluated the stiffness of FLLs using 2D-SWE [164,166,167,168,169,170]. HCC presents a large palette of stiffness values in 2D-SWE imaging, varying from 19.6 kPa in the 1 case included by Ronot et al. [164] to 44.8 kPa (range 15.8 kPa-97 kPa) in the Gerber study [170]. This variability can be explained by a multitude of factors, including lesion dimensions and the ROI positioning (peripheral—stiffer versus central—softer).

Furthermore, as objectified by Hwang et al. [171], the background liver plays an important role in the FLL diagnostic capability of 2D-SWE. In NAFLD patients, the liver can be fibrotic, which further hampers the stiffness measurement of FLLs, making it difficult to evaluate a malignant lesion versus a benign lesion. In the same phantom study by Hwang et al., the inclusion’s (FLL mimic) stiffness was increased when the inclusion was engulfed in a stiffer background, e.g., an FLL on a fibrotic liver [171]. A study by Grgurevic et al. concluded that a comprehensive 2D-SWE approach—defined as the statistical analysis of FLL stiffness, FLL to non-infiltrated liver stiffness ratio, as well as the intralesional variation of stiffness—would be able to differentiate between malignant FLLs and benign FLLs in 96% of patients [169]. In general terms, benign FLLs present as softer than their malignant counterparts [167,170].

Moreover, a recent study by Wang et al. [168] used an ultrasomics technique to investigate the possibility of discriminating malignant FLLs from benign FLLs through 2D-SWE. The team calculated an ultrasomics score (generated by a support vector machine from 15 ultrasomics features that were statistically obtained by Spearman correlation), as well as a combined score (generated by analyzing 4 SWE measurements and 15 ultrasomics features), to identify the method with the highest statistical accuracy. The authors focused their FLL study on two separate ROI point measurements (one peripheral and one central), to increase the measurement accuracy, as the literature reports different stiffness values in different points of the same FLL. Both the ultrasomics score and the combined score presented advantages compared to conventional 2D-SWE in differentiating malignant FLLs from benign FLLs with 0.96 AUC for both scores in the training cohort, as well as 0.91 AUC and 0.94 AUC, respectively, in the validation cohort. Furthermore, the combined score showed better diagnostic performance compared to the ultrasomics score and SWE measurements alone, suggesting a great potential of the ultrasomics method in discriminating between malignant FLLs and benign FLLs [168]. Table 6 presents the mean FLL stiffness values measured by 2D-SWE with the associated cut-off values for differentiating malignant FLLs from benign FLLs.

Two 2D-SWE studies by Guibal et al. [167] and Ronot et al. [164] found no significant differences between malignant and benign FLL stiffness. However, these studies had considerable limits. Guibal et al. suggested that a single diagnostic threshold would not present clinical value to discriminate between malignant and benign FLLs [167]. Ronot et al. included only a small percentage of patients with malignant lesions, which can cause a statistical bias in sampling [164]. There are several other limitations that must be mentioned. First and foremost, this technique cannot evaluate lesions situated over the general SWE limit—8 cm from the skin. Another patient-related limitation is connected to poor image acquisition, due to poor intercostal window and patient’s inability to hold the respiration when prompted [168]. Last, but not least, we mention the limitations that may appear in regard to the study design (inclusion/exclusion criteria), the heterogeneity of the lesions (e.g., the heterogeneity of different types of metastases), the operator’s experience with 2D-SWE, and the value overlaps. Although the reviewed papers present a great potential of 2D-SWE in characterizing malignant lesions, including HCC on a NAFLD-affected liver, further studies are required to evaluate the accuracy of this method and set specific cut-off values.

## 8. Conclusions

The rapidly growing prevalence of NAFLD and the implied higher risk of HCC development prompt for new diagnostic tools for both NAFLD and the NAFLD-related HCC. By non-invasive, disease-specific surveillance tools, such as US elastography, a subset of the non-cirrhotic NAFLD patients with a risk for developing HCC can be detected early, leading to a better outcome.

As a rather new and rapidly expanding field in hepatology, US elastography possesses many advantages in characterizing both diffuse and focal liver pathologies. This ultrasound-based method adds a new dimension to the characterization of the background liver and the FLL. Moreover, US elastography provides a rapid, non-invasive and inexpensive method for the clinician to evaluate liver steatosis (using CAP measurement) and fibrosis (using liver stiffness measurement), thus adding a new dimension to the conventional US examination of the background liver. Possessing both diagnostic and prognostic capabilities, US elastography contributes to better surveillance of the underlying liver disease. Furthermore, the development of new elastographic techniques, such as pSWE and 2D-SWE, opened the possibility of evaluating FLLs’ stiffness, providing a new category of data that may help in distinguishing between malignant and benign lesions. A comprehensive 2D-SWE approach has been reported to be able to differentiate malignant FLLs from benign FLLs in 96% of cases. In general terms, multiple studies reported a pattern related to FLL stiffness—metastases > HCC > FNH > hemangiomas—that may guide the physician towards the next step of the clinical reasoning. However, the present state of the literature emphasizes the imperfection of this method as a diagnostic tool, as there are no standardized cut-off values for differentiating between malignant and benign liver lesions.

Current US elastography techniques present a number of drawbacks, including a maximum evaluation depth of 8 cm, sensitivity to motion factors, and in some cases, overlapping stiffness values between malignant and benign FLLs. Nevertheless, despite the existing drawbacks, our opinion is that US elastography brought a new and innovative method to characterize FLLs. While we objectified its potential, we find that further studies are required to investigate the accurate characterization of HCC in NAFLD patients, considering the existing technical and conceptual limitations of these elastographic methods. We propose that further studies should focus on the interrelation of the HCC lesion with the background liver and thoroughly characterize the potential intralesional heterogeneity of the HCC lesion, for a comprehensive view upon the existing pathology.

## Figures and Tables

**Table 1:** Summary of advantages and limitations of each quantitative ultrasonic elastography technology. The current clinical indications and the corresponding sensitivity and specificity are described as well, with the mention that studies are underway for several other liver pathologies, including NAFLD.

Elastographic Technique	Indications	Se (Min-Max)/Sp (Min-Max) according to the EASL-ALEH Guide [34]	Advantages	Limitations
VCTE	HCV: First-line assessment [34,35]	56–97%/32–96%	- Less expensive [66], point-of-care examination; - Easy to perform by non-specialized personnel after appropriate training [67]; - Good reproducibility; - The quality criteria are well-defined; - Good diagnostic accuracy for the stages of fibrosis in the liver; - Can assess steatosis using the Controlled attenuation parameter (CAP); - More research work was involved for VCTE in NAFLD patients, compared to the alternatives.	- Low quality in the presence of obesity, congestion, cholestasis, inflammation, and ascites [66,67] (the use of the XL probe reduces the limits in obese patients); - Requires dedicated device; - No image and guidance provided; - Operator and patient-related variability.
	HBV: Useful to identify cirrhotic patients	52–98%/38–98%		
	NAFLD: Can be used to exclude cirrhosis	67–100%/64–91%		
	ALD: Can be used to exclude cirrhosis	80–86%/83–91%		
Point quantification SWE	HCV: First-line assessment [35]	68–100%/70–98%	- Results are less affected by ascites, obesity [68]; - Provides anatomical information; images are provided by B-mode ultrasound conventional system; - Provides the possibility of choosing the ROI; - The accuracy of diagnosis is comparable to VCTE for the stages of fibrosis [69]; - Low operator dependence.	- More expensive; - The quality criteria are not well-defined; - Small ROI size compared to VCTE; - Quality influenced by cholestasis [70]; - Needs experience in B-mode ultrasound; - The method is less evaluated in the literature.
	HBV: Useful to identify cirrhotic patients	50–100%/70–92%		
Two-dimensional SWE (2D-SWE)	HCV: First-line assessment [35]	75.9–91.4%/88.2–90.8% ^1^	- Adjustable size of ROI, larger than VCTE and pSWE; - Provides real-time images; - Results are less affected by ascites, obesity [68]; - Provides both anatomical information and tissue stiffness, since B-mode ultrasound images are superimposed to the colored maps of the stiffness; - Low operator dependence; - The range of values is high (5–150 kPa) [69]; - The accuracy of diagnosis is comparable to VCTE for the stages of liver fibrosis [69].	- More expensive; - Needs experienced operator in B-mode ultrasound; - Low quality when depth below 4–5 cm [71]; - Results influenced by food intake [72]; - The method is less evaluated in the literature.
	HBV: Useful to identify those cirrhotic patients	50.7–81.5%/70.4–88.4% ^2^		

^1^ Se and Sp data available from [73]; ^2^ Se and Sp data available from [74]. VCTE, vibration controlled transient elastography; pSWE, Point shear wave elastography.

**Table 2:** Performance of liver stiffness (LS) cut-off values by VCTE for detecting different stages of liver fibrosis in NAFLD patients.

Fibrosis Stage		≥F1			≥F2			≥F3			≥F4		
Study		Cut-Off (kPa)	AUROC	Se/Sp (%)	Cut-Off (kPa)	AUROC	Se/Sp (%)	Cut-Off (kPa)	AUROC	Se/Sp (%)	Cut-Off (kPa)	AUROC	Se/Sp (%)
Eddowes et al. [81] (*n* = 373)		N/S			8.2 ^1^ 6.1 ^2^ 12.1 ^3^	0.77	71/70 90/38 44/91	9.7 ^1^ 7.1 ^2^ 14.1 ^3^	0.80	71/75 90/50 48/90	13.6 ^1^ 10.9 ^2^ 20.9 ^3^	0.89	85/79 91/70 59/90
Furlan et al. [82] (*n* = 59)		N/S			8.8 ^1^ 4.8 ^2^ 8.8 ^3^	0.77	51.2/94.4 90.2/50 51.2/94.4	6.7 ^1^ 6.2 ^2^ 10.5 ^3^	0.86	86.4/70.3 90.9/59.5 50/91.9	N/S		
Hsu et al. [83] (*n* = 230 *)		6.2	0.818	65.6/67.1	7.6	0.866	76.3/79.6	8.8	0.841	77.2/78	11.8	0.836	80/81
Siddiqui et al. [77] (*n* = 393)		4.9	0.74	90/31	8.6 ^1^ 5.6 ^2^ 11.9 ^3^	0.79	66/80 90/44 40/90	8.6 ^1^ 6.5 ^2^ 12.1 ^3^	0.83	80.74 90/47 52/90	13.1 ^1^ 12.1 ^2^ 14.9 ^3^	0.93	89/86 90/82 69/90
Wong et al. [65] (*n* = 496)	M probe	N/S			N/S			> 15 kPa	0.90	28.6/98.7	>15 kPa	0.87	46.9/95.5
	XL probe	N/S			N/S				0.80	31.3/96.5		0.86	48.6/93
Jiang et al. [84] (*n* = 1753 *)		N/S			N/S	0.85	77/80	N/S	0.92	79/89	N/S	0.96	90/91
Lee et al. [85] (*n* = 94)		N/S			7.4	0.757	62.5/91.7	8.0	0.870	82.6/84.9	10.8	0.882	91.7/81.2
Petta et al. [63] (*n* = 324)		N/S			8.5	0.808	N/S	10.1	0.861		N/S		
Xiao et al. [80] (*n* = 429 *)	M probe	N/S			5.8 6.65–7 7.25–11	0.83	91.7/57.4 74.1/68.8 65.7/84.5	6.95–7.25 7.6–8 8.7–9 9.6–11.4	0.87	69.2/66.3 88.9/77.2 83.3/78 80.1/89.9	7.9–8.4 10.3–11.3 11.5–11.95 13.4–22.3	0.92	96.5/77.7 87.7/86.3 77.5/88.8 78.2/90.8
	XL probe	N/S			4.8–8.2	0.82	75.8/64.8	5.7–9.3	0.86	75.3/74	7.2–16	0.94	87.8/82
Boursier et al. [86] (*n* = 452)		N/S			N/S	0.842	N/S	8.7	0.831	88.4/62.9	N/S	0.864	N/S
Cassinotto et al. [87] (*n* = 291)		N/S			6.2	0.82	90/45	8.2	0.86	90/61	9.5	0.87	92/62
Imajo et al. [88] (*n* = 142)		7	0.78	61.7/100	11	0.82	65.2/88.7	11.4	0.88	85.7/83.8	14	0.92	100/75.9
Pathik et al. [89] (*n* = 110)		N/S			9.1	N/S	N/S	12	0.91	90/80	20	N/S	90/80
Kwok et al. [90] (*n* = 854)		N/S			6.7–7.7	0.79–0.987	79/75	8–10.4	0.76–0.98	85/85	10.3–17.5	0.91–0.99	92/92
Kumar et al. [78] (*n* = 205)		6.1	0.82	78/68	7	0.85	77/78	9 ^5^ 7.8 ^2^ 11.2 ^3^	0.94	85/88 96/78 71/93	11.8 ^5^ 10.6 ^2^ 19.4 ^3^	0.96	90/88 100/82 70/98
Myers et al. [43] (*n* = 75)	M probe	N/S			7.8	0.86	82/78	N/S	0.87	N/S	22.3	0.88	80/91
	XL probe	N/S			6.4	0.85	81/66	N/S	0.90	N/S	16.0	0.95	100/91
Lupsor et al. [91] (*n* = 72)		5.3	0.879	86.1/88.9	6.8	0.789	66.67/84.31	10.2	0.978	100/96.87	N/S		
Wong et al. [92] (*n* = 246)		N/S			5.8 ^2^ 7 ^4^ 9 ^3^	0.84	91.1/50.3 79.2/75.9 52.5/91.7	7.9 ^2^ 8.7 ^4^ 9 ^3^	0.94	91.1/75.3 83.9/83.2 75/91.6	10.3 ^2^ 10.3 ^4^ 11.4 ^3^	0.95	92/87.8 92/87.8 76/91
Yoneda et al. [76] (*n* = 97)		5.9	0.93	86.1/88.9	6.65	0.865	88.2/73.9	9.8	0.904	85.2/81.4	17.5	0.991	100/96.6

* meta-analysis, N/S = not specified. ^1^ Youden’s Index, YI; ^2^ Se > 90%; ^3^ Sp > 90%; ^4^ max diagnostic accuracy, DA; ^5^ Se + Sp max.

**Table 3:** Performance of ARFI for detecting different stages of liver fibrosis in NAFLD patients.

Fibrosis Stage	≥F2			≥F3			≥F4		
Study	Cut-Off (m/s)	AUROC	Se/Sp (%)	Cut-Off (m/s)	AUROC	Se/Sp (%)	Cut-Off (m/s)	AUROC	Se/Sp (%)
Lin et al. (*n* = 1147 ^1^) [105]	1.3	0.89	85/83	2.06	0.94	90/90	1.89	0.94	90/95
Jiang et al. (*n* = 982 ^1^) [84]	N/S	0.86	70/84	N/S	0.94	89/88	N/S	0.95	89/91
Lee et al. (*n* = 94) [85]	1.35	0.657	46.2/93.2	1.43	0.873	70/93.7	1.50	0.92	75/90.7
Cassinotto et al. (*n* = 291) [87]	0.95 1.32	0.77	90/36 56/91	1.15 1.53	0.84	90/63 59/90	1.3 2.04	0.84	90/67 44/90
Cui et al.(*n* = 125) [110]	1.34	0.848	81.8/78.3	1.34	0.896	95.2/74	2.48	0.862	77.8/93.1
Fierbinteanu et al. (*n* = 64) [101]	1.165	0.944	84.8/90.3	1.48	0.982	86.4/95.2	1.635	0.984	91.7/92.3
Cassinotto et al. (*n* = 321) [111]	1.38	0.81	71/78	1.57	0.85	75/80	1.61	0.88	74/78
Friedrich-Rust et al. ^2^ (*n* = 57) [68]	N/S	0.66	N/S	N/S	0.71	N/S	N/S	0.74	N/S
Osaki et al. (*n* = 23 ^3^) [103]	1.79 ± 0.78	N/S	N/S	2.20 ± 0.74	N/S	N/S	2.90 ± 1.01	N/S	N/S
Yoneda et al. (*n* = 54) [112]	N/S			1.77	0.93	100/91	1.90	0.937	100/96

^1^ meta-analysis, ^2^ ARFI measurement for the right lobe, ^3^ NASH patients.

**Table 4:** Performance of LSM was assessed by 2D-SWE for detecting different stages of liver fibrosis in patients with NAFLD.

Fibrosis Stage	≥F1			≥F2			≥F3			≥F4		
Study	Cut-Off (kPa)	AUROC	Se/Sp (%)	Cut-Off (kPa)	AUROC	Se/Sp (%)	Cut-Off (kPa)	AUROC	Se/Sp (%)	Cut-Off (kPa)	AUROC	Se/Sp (%)
Lee et al. (*n* = 102) [108]	6.3	0.82	63/88	7.6	0.87	89/77	9.0	0.95	100/85	N/S		
Herrmann et al. (*n* = 156) [113]	N/S			7.1	0.855	N/S	9.2	0.928	N/S	13.0	0.917	N/S
Takeuchi et al. (*n* = 71) [109]	6.61	0.82	79/67	11.57	0.75	52/44	13.07	0.82	63/57	15.73	0.90	100/82
Lee et al. (*n* = 94) [85]	N/S			8.3	0.759	87/55.3	10.7	0.809	90/61.2	15.1	0.906	90/78
Xiao et al. (*n* = 429 ^1^) [80]	N/S			2.67–9.4	0.89	85/94.4	3.02–10.6	0.91	89.9/91.8	3.36	0.97	100/85.6
Cassinotto et al. (*n* = 291) [87]	N/S			6.3 ^3^ 8.7 ^4^	0.86	90/50 ^3^ 71/90 ^4^	8.3 ^3^ 10.7 ^4^	0.89	91/71 ^3^ 71/90 ^4^	10.5 ^3^ 14.5 ^4^	0.88	90/72 ^3^ 58/90 ^4^
Ochi et al. (*n* = 181) [114]	2.47 ^2^	0.838	0.649/0.969	2.76 ^2^	0.853	86/88.6	3.02 ^2^	0.878	88.2/91.5	3.36 ^2^	0.965	100/85.6

^1^ meta-analysis, ^2^ study used elastic ratio, ^3^ for Se ≥ 90%, ^4^ for Sp ≥ 90%.

**Table 5:** A collection of shear wave velocity values (mean in m/s, range) for a predefined number of focal liver lesions (FLLs) in different studies, using the pSWE technology. The table includes the SWV cut-off values (m/s) for discriminating malignant versus benign FLLs, their corresponding sensitivity (Se) and specificity (Sp), as well as the statistical interpretation of the discrimination of HCC lesions from others.

Study	Cut-Off Value Malignant Versus Benign (m/s)	Se/Sp (%)	HCC	Metastases	Hemangiomas	FNH	Hepatocellular Adenoma	Statistically Significant/Not Significant Difference between SWV of HCC and Other FLLs
Park et al. [139]	1.82	71.8/75	2.48 ± 0.84 (*n* = 24)	2.35 ± 1.18 (*n* = 8)	1.83 ± 0.62 (*n* = 5)	0.97 ± 0.48 (*n* = 3)	N/S	Significant difference: HCC—benign lesions (*p* = 0.006)
Akdogan et al. [140]	2.32	93/60	2.75 ± 0.53 (*n* = 10)	3.59 ± 0.51 (*n* = 22)	2.15 ± 0.73 (*n* = 34)	3.22 ± 0.18 (*n* = 4)	N/S	No significant difference: HCC—hemangiomas (*p* > 0.05) Significant difference: HCC—metastatic lesions (*p* < 0.05)
Kim et al. [141]	2.73	96.4/65.8	2.66 ± 0.94 (*n* = 26)	2.82 ± 0.96 (*n* = 24) with colon cancer metastasis 3.70 ± 0.61 (*n* = 20)	1.80 ± 0.57 (*n* = 28)	N/S	N/S	No significant difference: HCC—hemangiomas (*p* > 0.05)
Davies et al. [142]	2.5	97.1/100	N/S	4.23 ± 0.59 (*n* = 10)	1.35 ± 0.48 (*n* = 35)	N/S	N/S	N/S
Gallotti et al. [143]	N/S	N/S	2.17 ± 0.85 (*n* = 6)	2.87 ± 1.13 (*n* = 9)	2.30 ± 0.95 (*n* = 7)	2.75 ± 0.95 (*n* = 13)	1.25 ± 0.37 (*n* = 5)	No significant difference: HCC—hemangiomas. Significant difference: HCC—adenomas (*p* < 0.05)
Frulio et al. [144]	N/S	N/S	2.4 ± 1.01 (*n* = 24)	3.0 ± 1.36 (*n* = 12)	2.14 ± 0.49 (*n* = 15)	3.14 ± 0.63 (*n* = 19)	1.90 ± 0.86 (*n* = 9)	No significant difference: malignant—benign groups (*p* N/S).
Dong et al. [145]	2.06	80.6/88	2.63 (range 1.84–5.68) (*n* = 104)	2.78 (range 1.02–3.15) (*n* = 11)	1.5 (range 0.79–2.61) (*n* = 11)	1.35 (range 0.69–2.94) (*n* = 5)	N/S	Significant difference: Malignant—benign lesions (*p* < 0.05)
Guo et al. [146]	2.13	83.3/77.9	3.07 ± 0.89 (*n* = 24)	2.74 ± 1.06 (*n* = 26)	1.48 ± 0.70 (*n* = 47)	2.30 ± 1.18 (*n* = 7)	N/S	Significant difference: HCC—hemangiomas (*p* < 0.001) Significant difference: HCC—focal fatty degeneration (not mentioned in the current table, *p* = 0.006)
Zhang et al. [147]	2.16	81.3/74.1	2.59 ± 0.91 (*n* = 61)	3.20 ± 0.62 (*n* = 39)	1.33 ± 0.38 (*n* = 28)	1.90 ± 0.45 (*n* = 14)	N/S	Significant difference: Malignant—benign lesions (*p* < 0.01)
Yu et al. [148]	2.72	69/89	2.49 ± 1.07 (*n* = 28)	2.73 ± 0.89 (*n* = 13)	1.75 ± 0.80 (*n* = 35)	2.18 ± 0.84 (*n* = 15)	1.79 ± 0.14 (*n* = 2)	Significant difference: HCC—benign lesions (*p* < 0.01, overlap) Significant difference: HCC—hemangiomas (*p* < 0.01)
Heide et al. [149]	N/S	N/S	2.63 ± 1.09 (*n* = 5)	2.88 ± 1.16 (*n* = 17)	2.36 ± 0.77 (*n* = 13)	3.11 ± 0.93 (*n* = 17)	2.23 ± 0.97 (*n* = 2)	No significant difference: Malignant—benign lesions (*p* = 0.23).
Galati et al. [150]	2.0	74.6/80.7	2.47 ± 1.425 (*n* = 39)	3.29 ± 1.2325 (*n* = 28)	1.34 ± 0.9125 (*n* = 52)	N/S	N/S	Significant difference: Malignant lesions—hemangiomas (*p* N/S)
Cho et al. [151]	2.0	74/82	2.45 ± 0.81 (*n* = 17)	2.18 ± 0.96 (*n* = 8)	1.51 ± 0.71 (*n* = 17)	N/S	N/S	Significant difference: HCC—hemangiomas (*p* < 0.05)
Wu et al. [152]	2.22	51.9/85.7	Malignant: 2.25 ± 0.80 (*n* = 27)		Benign: 1.70 ± 0.58 (*n* = 28)			Significant difference: Malignant—benign lesions (*p* = 0.007)
Shuang-Ming et al. [153]	2.22	89.7/95	Malignant: 3.16 ± 0.80 (*n* = 68)		Benign: 1.47 ± 0.53 (*n* = 60)			Significant difference: Malignant—benign lesions (*p* < 0.001)
Kapoor et al. [154]	2.5	88/83	2.4 (range 1.28–3.5) (*n* = 7)	3.28 (range 2.9–3.65) (*n* = 18)	Benign: 1.83 (range 1.26–2.39) (*n* = 15)			Significant difference: HCC—metastatic nodules (*p* = 0.008)

**Table 6:** Mean stiffness values (kPa) of FLLs measured by 2D-SWE with the associated cut-off values to differentiate malignant FLLs from benign FLLs.

Study	Cut-Off Value Malignant Versus Benign	Se/Sp (%)	HCC	Metastases	Hemangiomas	FNH	Hepatocellular Adenoma	Statistically Significant/Not Significant Difference between Stiffness of HCC and Other FLLs
Tian et al.^1^ [166]	39.60	87.74/83.67	61.83 ± 28.87 (*n* = 103)/Parenchyma: 15.94 ± 7.37	90.32 ± 54.71 (*n* = 35)/Parenchyma: 10.93 ± 36.64	20.56 ± 10.74 (*n* = 37)/Parenchyma: 9.04 ± 2.44	38.72 ± 18.65(*n* = 15)/Parenchyma: 9.09 ± 2.64	N/S	Significant difference: Intrahepatic cholangiocarcinomas—HCC (*p* < 0.0001) Significant difference: metastases—HCC (*p* = 0.0237) Significant difference: malignant—benign lesions (*p* < 0.001) Significant difference: HCC—FNHs (HCC > FNH, *p* = 0.0012)
Guibal et al. [167]	N/S	N/S	14.86 ± 10 (*n* = 26)	28.8 ± 16 (*n* = 53)	13.8 ± 5.5 (*n* = 22)	33 ± 14.7 (*n* = 16)	9.4 ± 4.3 (*n* = 10)	Significant difference: HCC—cholangiocarcinomas (*p* = 0.0004) Significant difference: HCC—metastases (*p* = 0.0059)
Wang et al. [168]	25.76 (Emean), 0.85 (combined score)	92.59/87.50 (combined score)	39.31 ± 12.50 (*n* = 83)	56.99 ± 33.13 (*n* = 24)	13.71 ± 9.24 (*n* = 33)	30.56 ± 11.86 (*n* = 11)	N/S	N/S
Ronot et al. [164]	N/S	N/S	19.6 (*n* = 1)	N/S	17.1 ± 7 (*n* = 20)	33.3 ± 12.7 (*n* = 60)	19.7 ± 9.8 (*n* = 17)	No significant difference: Malignant—benign lesions (*p* = 0.64)
Grgurevic et al. [169]	22.3	83/86	29.57 ± 11.67 (*n* = 57)	37.93 ± 10.61 (*n* = 94)	14.10 ± 6.44 (*n* = 71)	30.51 ± 32.05 (*n* = 20)	N/S	Significant difference: Malignant—benign lesions (*p* < 0.001)
Gerber et al. [170]	20.7	79.7/62	44.8 (range 15.8–97) (*n* = 16)	29.5 (range 4.1–142.9) (*n* = 41)	16.35 (range 5.4–71.9) (*n* = 18)	16.55 (range 2.1–69.7) (*n* = 18)	8.9 (*n* = 1)	Significant difference: Malignant—benign lesions (*p* < 0.0001) Significant difference: Cholangiocarcinomas—HCC (*p* = 0.033) Significant difference: Cholangiocarcinomas—metastases (*p* = 0.0079)

^1^ only maximal stiffness values presented within the paper.
